# Complete regression of primary cutaneous malignant melanoma associated with distant lymph node metastasis: a teaching case mimicking blue nevus

**DOI:** 10.1186/s13104-016-2174-4

**Published:** 2016-07-26

**Authors:** Sohsuke Yamada, Aya Nawata, Manabu Yoshioka, Tsubasa Hiraki, Michiyo Higashi, Kazuhito Hatanaka, Akihide Tanimoto

**Affiliations:** 1 Department of Pathology, Field of Oncology, Kagoshima University Graduate School of Medical and Dental Sciences, Kagoshima, Japan; 2 Department of Pathology and Cell Biology, School of Medicine, University of Occupational and Environmental Health, Kitakyushu, Japan; 3 Department of Dermatology and Immunology, School of Medicine, University of Occupational and Environmental Health, Kitakyushu, Japan

## Abstract

**Background:**

Malignant melanoma (MM) tends to be spontaneously regressed, however, complete regression of primary cutaneous MM is an extremely rare phenomenon. Our aim is to be aware that pathologists and/or dermatologists can readily misinterpret it as the other benign or malignant lesions.

**Case presentation:**

A gradually growing and verrucous hypopigmented macule had been noticed in the right sole of a 65-year-old Japanese male since 2 years before, and it turned to be a solitary bluish to black patch with surrounding depigmentation and was recently decreased in size. In parallel, the patient had a rapidly growing black-pigmented mass lesion at the right inguen. The cutaneous specimen from the sole showed an aggregation of many melanophages predominantly in the middle to deep layer of dermis, associated with surrounding fibrosis, reactive vascular proliferation and CD8-positive T-lymphocytic infiltrate, covered by attenuated epidermis with absence of rete ridge. However, no remnant MM cells were completely seen in the step-serial sections. We first interpreted it as blue nevus. By contrast, the inguinal mass revealed a diffuse proliferation of highly atypical mono- to multi-nucleated large cells having abundant eosinophilic cytoplasm in the enlarged lymph node tissue. Immunohistochemical findings demonstrated that these atypical cells were specifically positive for HMB45 and Melan A. Therefore, we finally made a diagnosis of complete regression of primary cutaneous MM associated with distant lymph node metastasis of MM.

**Conclusion:**

Careful, not only general/cutaneous but histopathological, examinations should be necessary and adjunctive aids for reaching the correct diagnosis of complete regression of cutaneous MM.

## Background

Among all malignant melanomas (MMs), partial regression is not uncommon, accounting for 10 to 35 % regardless of Breslow’s tumor thickness [1, 2]. In particular, thin MMs, less than 1 mm thickness, reportedly show partial regression in up to 61 % of all tumor lesions [2, 3]. By contrast, complete regression of primary cutaneous MM is a much rarer occurrence with merely 0.24 % in 2464 MM patients [4], and only 39 cases have been reported in the English-language literature, to the best of our knowledge [2]. Smith and Stehlin [5] have already established criteria for complete regression of MM as follows: (a) history and/or clinical evidence of a pigmented lesion situated in an area drained by tumor-involved lymph nodes; (b) absence of any other primary lesions identifiable by history or physical examination that could represent the origin of MM; (c) presence of atypical pigmented or depigmented change in the skin at the site of the untreated presumed primary lesion, with all or a majority of the typical histologic features associated with regression found on biopsy (attenuated epidermis, dermal melanophages, lymphocytic or chronic inflammatory infiltrate, reactive vascular proliferation, and fibrosis); and (d) Histologic absence of MM cells in excised lesions (step-serial sections of the entire primary site are important to confirm the absence of small residual foci of malignant cells) [5, 6]. Clinically, complete regression of MM is characterized by variability with a ‘change’, manifesting as hyper- to hypo-pigmented macules, patches, papules, and plaques, measuring from 0.4 to 3.0 cm in diameter, and most commonly displaying enlargement, friability, bleeding, and eventual regression [2]. These changes have a tendency to occur from 2 months to 14 years before the diagnosis of metastatic MM [2]. Moreover, it has been reported that the patients’ prognoses are highly variable, ranging from 6 weeks to 11 years after its diagnosis, whereas 13 patients actually died of MM progression with an average survival of 13 months [2, 5, 6]. Thus, it is critical to establish an accurate initial diagnosis, including whether associated metastatic MM sites or not in the whole body by thorough clinicopathological examinations.

We report an extremely rare case of complete regression of primary cutaneous MM coexisted with distant lymph node metastasis, possibly confused with other benign or malignant lesions of the skin.

## Case presentation

### Methods

The patient was a 65-year-old Japanese man. The cutaneous and inguinal specimens after fixation in 10 % neutral buffered formalin were embedded in paraffin for histological or immunohistochemical examinations. Furthermore, we prepared more than 10 step-serial H&E staining sections of the entire primary site (right sole), and absolutely confirmed the absence of residual foci of MM cells [2, 5, 6]. All immunohistochemical stainings were carried out using Dako Envision kit (Dako Cytomation Co., Glostrup, Denmark) according to the manufacturer’s instructions. [7–11]. We counted the numbers of CD4 (Dako, diluted 1:1)—and CD8 (Nichirei Biosciences Inc., Tokyo, Japan, diluted 1:1)—positive infiltrating T-lymphocytes in the cutaneous pigmented lesion (right sole) in 10 randomly selected fields of per section (original magnification: ×400) [7–11].

All histological and immunohistochemical slides were evaluated by two independent observers (certified surgical pathologists in our department) [7–11]. Agreement between observers was excellent (>0.9) for all antibodies investigated as measured by interclass correlation coefficient. For the few instances of disagreements, a consensus score was determined by the third board-certified pathologists in our department [7–11]. All values are expressed as the mean ± SE. Significant differences were analyzed using Student’s *t* test. Values of *P* < 0.05 were considered to be statistically significant.

### Clinical summary

The patient had an unremarkable previous medical history, except for essential hypertension. The patient was admitted to hospital due to an approximately 2-months history of inguinal growing mass and pain. There had been no history of malignancy, immunosuppressive disorders, use of immunosuppressive medications, or unusual infections, including acquired immunodeficiency syndrome (AIDS).

**Fig. 1:**
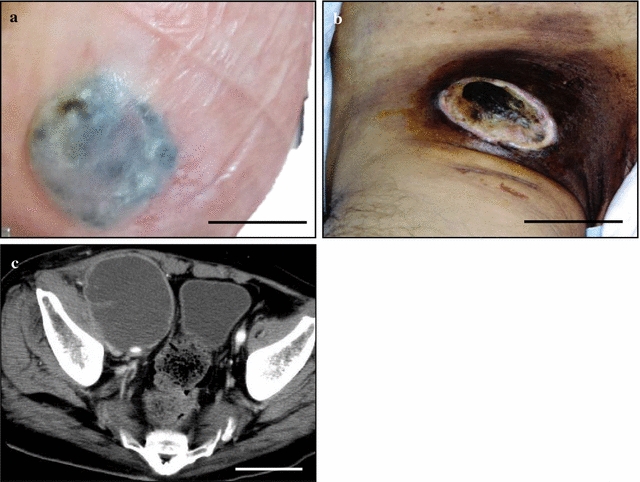
Macroscopic and imaging findings of this complete regression of cutaneous MM on the right sole associated with right inguinal lymph node metastasis. **a**, **b** The patient had a solitary bluish to *black patch* with surrounding depigmentation, accompanied by recent decrease in size, measuring up to 20 mm in the *right sole*. In fact, he had noticed it as a gradually growing, hemorrhagic and verrucous hypopigmented macule since approximately 2 years before. *Bar*  2 cm. **b** In parallel, he suffered from a rapidly growing and painful black-pigmented mass lesion with surface skin ulcer at the right inguen especially for recent 2 months, partly extending to the right scrotum and thigh. *Bar*  5 cm. **c** A pelvic CT scan revealed a relatively well-defined huge and low-density mass with peripheral enhancement, measuring approximately 10 × 8 cm. *Bar*  5 cm

He had noticed a gradually growing, hemorrhagic and verrucous hypopigmented macule since approximately 2 years before, and it turned to be a solitary bluish to black patch with surrounding depigmentation, accompanied by recent decrease in size, measuring up to 20 mm in the right sole (Fig. 1a). There were no other primary cutaneous lesions identifiable by his history or careful physical examination that could represent the original MM. In parallel, he suffered from a rapidly growing and painful black-pigmented mass lesion with surface skin ulcer at the right inguen especially for recent 2 months (Fig. 1b), partly extending to the right scrotum and thigh (Fig. 1b). A pelvic CT scan showed a relatively well-defined huge and low-density mass with peripheral enhancement, measuring approximately 12 × 10 cm (Fig. 1c). CT scans of the head, neck, chest and abdomen disclosed no definite evidence of neoplastic foci or other metastases in the lymph nodes or other organs. Laboratory data, including blood cell count, chemistry and tumor markers, were within normal limits. Within the post-operative 2 years with additional treatment of chemotherapy, including dacarbazine, nimustine, cisplatin, tamoxifen, (DAC-tam) and interferon-β, recurrence of small (less than 5 mm) MMs in situ was noted in the right foot, waist and back at 1 month after the operation. But, surprisingly, the patient had neither local invasion nor metastases of the other sites, respectively, and he was alive and well.

### Pathological findings

**Fig. 2:**
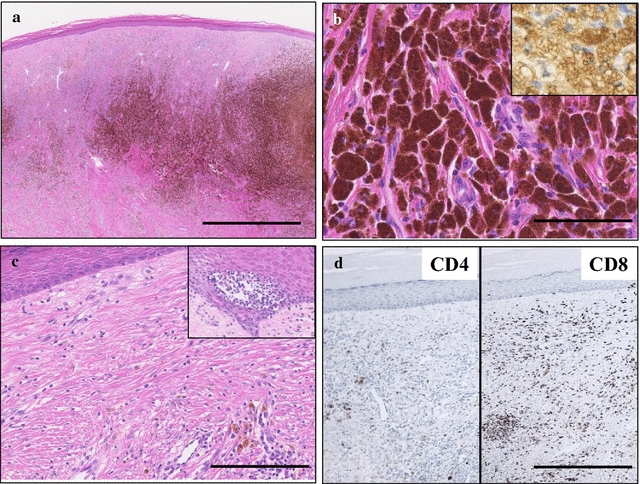
Microscopic examination of the complete regression of primary cutaneous MM. **a** On low-power view of the surgical cutaneous specimen from the right sole, a hyperpigmented lesion was made up of an aggregation of melanophages with surrounding variably sclerotic fibrosis mainly in the middle to deep layer of dermis (H&E stains). The covering non-disordered epidermis exhibited attenuated and atrophic change with absence of rete ridge. *Bar*  2 mm. **b** On high-power view (H&E stains), these aggregated round to oval cells contained bland-looking small nuclei and abundant melanin-pigmented cytoplasm. In addition, immunohistochemically, these melanophages were strongly positive for CD68 (inset). *Bar*  50 μm. **c** In these fibrotic dermis, reactive vascular networks were noted, associated with mild interstitial and perivascular lymphocytic infiltrate. Furthermore the peripheral epidermis displayed exocytosis of focal nested lymphocytes (*inset*). *Bar*  500 μm. **d** In immunohistochemistry, dermal infiltrating CD8-positive T-lymphocytes (*right*) were markedly larger than CD4-positive T-lymphocytes (*left*) in number in this cutaneous lesion. *Bar*  1000 μm

The surgical cutaneous specimen from the right sole (Fig. 2a) showed a hyperpigmented lesion made up of an aggregation of melanophages with surrounding variably sclerotic fibrosis mainly in the middle to deep layer of dermis. On high-power view, these aggregated round to oval cells had bland-looking small nuclei and abundant melanin-pigmented cytoplasm (Fig. 2b). In addition, reactive vascular networks were occasionally seen in these fibrous stroma, associated with mild interstitial and perivascular lymphocytic infiltrate (Fig. 2c). The covering non-disordered epidermis exhibited attenuated and atrophic change with absence of rete ridge, carrying no in situ MM cells within our thorough examination (Fig. 2a, c). Interestingly, the peripheral epidermis sometimes showed exocytosis of focal nested lymphocytes (Fig. 2c). Nevertheless, there was no MM cells infiltration from the epidermis to dermis, subcutis in all of the prepared step-serial H&E staining sections. Based on these features, we first diagnosed it as a benign cutaneous lesion, such as blue nevus. Immunohistochemically, these melanophages were strongly positive for CD68 (KP-1; Dako, diluted 1:100) (Fig. 2b), whereas completely negative for S-100 protein (Dako, diluted 1:900), HMB45 (Enzo Life Sciences Ltd., New York, diluted 1:100), and Melan A (NOVOCASTRA, 1:50) (data not shown). Furthermore, we counted the numbers of CD4- and CD8-positive infiltrating T-lymphocytes in this cutaneous lesion (Fig. 2d). Intriguingly, CD8-positive T-lymphocytes were markedly larger than CD4-positive lymphocytes in number (CD4: 1.8 ± 0.5 per 1 high power field vs. CD8: 54.6 ± 5.2 per 1 high power field; *P* < 0.0001) (Fig. 2d).

**Fig. 3:**
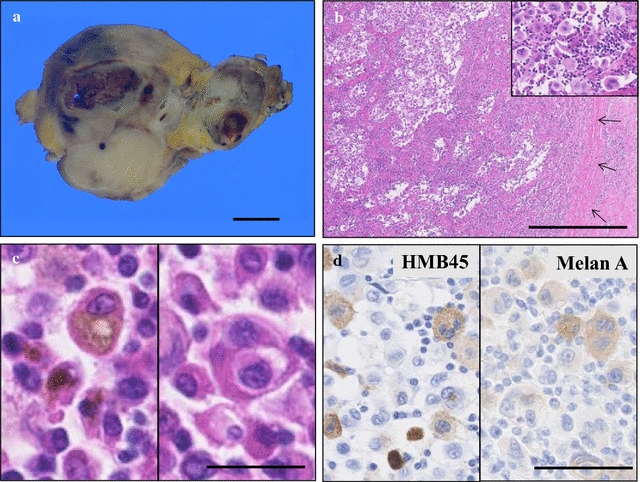
Gross and microscopic examination of the metastatic lymph node metastasis. **a** On gross findings of the surgical specimen from the inguinal to scrotal and femoral mass, the huge, relatively well-demarcated and multi-nodular tumor, measuring approximately 8 × 6 cm, showed *gray-whitish* to *yellow-whitish* cut surfaces with hemorrhagic and yellowish necrotic foci. *Bar*  1 cm. **b** Microscopically, this inguinal tumor consisted of a diffuse proliferation of markedly atypical large cells, appearing predominantly as single cells (*inset*), involving and erasing the remarkably enlarged pre-existing lymph node tissue, partly surrounded by fibrous capsule (*arrows*).* Bar*  1000 μm. **c** On high-power view, these tumor cells contained hyperchromatic, pleomorphic mono- to multi-nuclei, and abundant eosinophilic to sometimes clear cytoplasm, admixed with a number of lymphocytes. Additionally, the large tumor cells occasionally had melanin pigments in the cytoplasm (*left*) and often showed autophagy (*right*).* Bar*  50 μm. **d** In immunohistochemistry, these malignant cells were specifically positive for melanocytic markers, such as HMB45 (*left*) and Melan A (*right*). *Bar*  100 μm

Next, gross examination of the surgical specimen from the inguinal to scrotal and femoral mass showed that the huge, relatively well-demarcated and multi-nodular tumor, measuring approximately 13 × 11 × 7 cm, had gray-whitish to yellow-whitish cut surfaces with hemorrhagic and yellowish necrotic foci (Fig. 3a). Microscopically, the inguinal tumor consisted of a diffuse proliferation of markedly atypical large cells (Fig. 3b) having hyperchromatic, pleomorphic mono- to multi-nuclei, and abundant eosinophilic to sometimes clear cytoplasm, appearing predominantly as single cells, admixed with a large number of likely pre-existing lymphocytes (Fig. 3c). These proliferating tumor cells involved and erased the remarkably enlarged lymph node tissue, partly surrounded by fibrous capsule (Fig. 3b). On high-power view, the large tumor cells occasionally had melanin pigments in the cytoplasm and often showed autophagy (Fig. 3c). In immunohistochemistry, these malignant cells were specifically positive for melanocytic markers, such as HMB45 (Fig. 3d) and Melan A (Fig. 3d), and S-100 protein (data not shown), whereas negative for CD68 (data not shown).

Based on all the clinicopathological features, we confirmed that these proliferating atypical cells were derived from MM, and finally made a diagnosis of complete regression of primary cutaneous MM on the right sole, associated with distant inguinal lymph node metastasis of MM.

## Conclusion

The most important histological differential diagnosis in the present completely regressed MM case is with a remnant MM of the skin. Immunohistochemical analyses would be able to resolve the distinction from the malignancy easily, because the highly atypical MM cells were specifically positive for melanoma-associated antigens, representing as HMB45 and Melan A, whereas completely negative for histiocytic markers, such as CD68, but in striking contrast, the bland-looking melanophages were not, as shown in the current report. Moreover, as previously indicated by Smith and Stehlin [5], after we have prepared more than 10 step-serial H&E-stained sections of the entire primary site from the right sole, we can completely confirm the absence of residual foci of the MMs. Besides, our case actually fitted the clinicopathological categories for complete regression of MM as follows: (i) history and clinical evidence of this pigmented lesion on the right sole situated in an area drained by the MM-involved right inguinal lymph nodes; (ii) complete absence of any other primary lesions identifiable by history or physical examinations that could represent the original sites of MM; (c) presence of variable pigmented and/or depigmented change in the right sole, as the untreated primary cutaneous lesion, accompanied by all of the typical, histopathologically regressed features, including attenuated atrophic epidermis, dermal aggregation of many melanophages, lymphocytic infiltrate, reactive vascular proliferation, and surrounding fibrosis [2, 5, 6]. On the other hand, among benign lesions, pathological differential diagnoses would include blue nevus or scar, since complete regression of cutaneous MM and each benign lesion can share variable histological features, as described here. Despite that, we pathologists and/or dermatologists should be aware that thorough and adequate clinicopathological examinations can readily distinguish from the above malignant to benign diagnoses.

As to the present case, it is clinicopathologically remarkable for two reasons at least: first, within our thorough investigation, this is the first single-case report of complete regression of cutaneous MM arising in the sole, clinicopathologically mimicking blue nevus, due to histologically middle to deep dermal melanophages at least in part. Indeed, to date, the papers reported as completely regressed MM have been very rarely published in the English literatures, but the number of its ‘true’ cases remain unclear, because metastatic MM of unknown primary origin accounts for approximately 2.5 to 10 % among all presenting MMs, according to most large series of MM [2, 12]. Furthermore, it is very likely that most cases of undetected primary cutaneous MMs undergo near-complete regression prior to metastatic involvement and progression [12]. In that sense, it is suggested that complete regression of primary cutaneous MM might be much more common than generally considered. Second, the present primary site surrounded by the variable fibrosis and vascular networks contained a dermal infiltration of much more predominant CD8-positive T-lymphocytes than CD4-positive ones, as shown in Fig. 2d. According to one possible theory, ‘antitumor immune response’, explaining the MM regression mechanism(s) [12, 13], MM cells can express specific antigens, including Melan A (MART-1), identified by the distinct immune systems of each MM patient. Subsequently, MM patients can generate spontaneous immune responses to a wide range of these MM-specific antigens, via tumor-infiltrating lymphocytes (TILs), especially CD4- and CD8-positive T-lymphocytes [14]. In particular, it is possible that the cell-mediated cytotoxicity of CD8-positive T-lymphocytes infiltrate can serve as an early sign of MM regression, similar to the patients with halo nevi, as previously demonstrated [2, 15]. Nevertheless, future convincing data will be further required to determine whether our speculation is significant or not.

We reported an extremely rare case of complete regression of primary cutaneous MM associated with distant lymph node metastasis of MM, dermatopathologically mimicking blue nevus at the primary site of MM. All pathologists and dermatologists should be aware that its characteristic features could easily lead to a misdiagnosis of the other benign or malignant lesions. Furthermore, it is suggested that we pathologists and/or dermatologists should be aware that thorough and adequate clinicopathological analyses, including careful general/cutaneous examinations and step-serial H&E staining sections of the entire primary site, are very useful and adjunctive aids for recognizing no evidence of malignancy (i.e., no residual MM cells) and reaching the correct diagnosis of completely regressed cutaneous MM associated with metastatic foci. In this context, the present case report should interest the scientific community, taken together with new histopathological findings and specific recommendations for diagnostic pathology.

## Abbreviation

MM: malignant melanoma
